# Association between increased levels of amyloid-β oligomers in plasma and episodic memory loss in Alzheimer’s disease

**DOI:** 10.1186/s13195-019-0535-7

**Published:** 2019-10-25

**Authors:** Xue Meng, Tao Li, Xiao Wang, Xiaozhen Lv, Zhiyu Sun, Jichun Zhang, Feng Su, Sungmin Kang, SangYun Kim, Seong Soo A. An, Xin Yu, Chen Zhang, Huali Wang

**Affiliations:** 1 Dementia Care and Research Center, Beijing Key Laboratory for Translational Research on Diagnosis and Treatment of Dementia, Peking University Institute of Mental Health (Sixth Hospital), Beijing, 100191 China; 2 National Clinical Research Center for Mental Disorders, Key Laboratory for Mental Health, National Health Commission, Beijing, 100191 China; 3 State Key Laboratory of Membrane Biology, School of Life Sciences, PKU-IDG/McGovern Institute for Brain Research, Peking University, Beijing, 100871 China; 4 Department of Research and Development, PeopleBio, Inc., Seongnam-si, Gyeonggi-do Republic of Korea; 5 Department of Neurology, Seoul National University Bundang Hospital and Seoul National University College of Medicine, 82, Gumi-ro 173, Bundang-gu, Seongnam-si, Gyeonggi-do 463-707 Republic of Korea; 6 Department of Bionano Technology, Gachon University, Sujeong-gu, Seongnam-si, Gyeonggi-do 461-701 Republic of Korea

**Keywords:** Alzheimer’s disease, Amyloid oligomers, Plasma, Episodic memory

## Abstract

**Objective:**

The objectives of this study were to investigate whether the plasma levels of oligomeric amyloid-β (OAβ) were affected in Alzheimer’s disease (AD) and to examine the associations (or possible correlations) between plasma OAβ levels and memory performance.

**Method:**

Thirty subjects with AD and 28 cognitively normal controls were recruited in the study. The multimer detection system (MDS) was used to measure the levels of OAβ in the plasma. In addition to assessing the general cognitive function with the Mini-Mental State Examination (MMSE), Cognitive Abilities Screening Instrument (CASI), and Alzheimer’s Disease Assessment Scale–cognitive portion (ADAS-Cog), the common objects memory test (COMT) was used to examine the episodic memory performance. Pearson’s and partial correlation analyses were conducted to explore the associations between cognitive performance and OAβ levels in the plasma. A receiving operating curve (ROC) analysis was used to discriminate between the AD and control groups.

**Results:**

The plasma OAβ levels in the AD group were significantly higher than those in the control group [1.88 (0.38) ng/ml vs 1.20 (0.40) ng/ml, *p* < 0.001]. The elevated levels of plasma OAβ showed a strong correlation with cognitive performance in patients with AD, including an inverse correlation with scores on the MMSE (*r* = − 0.43, *p* = 0.02), CASI (*r* = − 0.56, *p* < 0.01), and the immediate recall (*r* = − 0.45, *p* = 0.01), 5-min delayed recall (*r* = − 0.56, *p* < 0.01), and 30-min delayed recall (*r* = − 0.71, *p* < 0.001) tests of the COMT, and a positive correlation with the ADAS-Cog scores (*r* = 0.59, *p* < 0.001). The EDTA plasma Aβ oligomer optical density (OD) value measured using the MDS could discriminate between the AD and control groups with an area under the curve (AUC) of 0.89. The optimal sensitivity and specificity were 82.1% and 90.0%, respectively.

**Conclusion:**

The elevated levels of OAβ in the plasma distinguished the AD and control groups and were associated with the severity of symptoms, especially memory performance, in patients with AD. Our results suggested that plasma OAβ could potentially be a simple and non-invasive blood-based biomarker for AD diagnosis. Furthermore, longitudinal studies are warranted to explore the application of plasma OAβ levels as a valid diagnostic biomarker in patients with AD.

**Electronic supplementary material:**

The online version of this article (10.1186/s13195-019-0535-7) contains supplementary material, which is available to authorized users.

## Introduction

The amyloid cascade hypothesis suggests that Alzheimer’s disease (AD) results from the imbalance between the production and clearance of amyloid-β (Aβ) [1]. The imbalance could lead to the accumulation and oligomerization of Aβ_42_ in the limbic and association cortices. Currently, Aβ oligomers are widely recognized as the most toxic and pathogenic forms of Aβ. The oligomerization of Aβ (OAβ) would be triggered from conformational alterations of the monomeric Aβ. The low-molecular-weight dimers and trimers could aggregate and form soluble oligomers and then high-molecular-weight oligomers, which would eventually evolve into insoluble fibrils. Previous experiments have shown that OAβ may trigger neuronal toxicity and synaptic loss. In the transgenic AD mouse model, OAβ levels in the brain, rather than total amyloid plaque accumulation, correlated closely with neuronal loss [2, 3]. Gulisano et al. found that OAβ could impair the long-term potentiation and spatial memory [4]. In addition, Hou et al. found that the inhibition of OAβ could improve spatial learning and memory function in PS1V97L transgenic mice [5]. These results implied that OAβ may serve as a potential fingerprint biomarker of memory function in AD [6, 7].

Results from several studies have revealed that the elevated OAβ levels in cerebrospinal fluid (CSF) correlated well with cognitive decline in patients with AD [8–10]. As the blood–brain barrier may break down in patients with AD, the levels of OAβ in the blood could reflect pathologic processes in the brains of AD patients [11, 12].

Although several sources of evidence have supported the notion that deficits in episodic memory manifest in patients with AD [13–16], it has remained unclear whether plasma OAβ levels would correlate with memory function in AD. Few studies have explored the potential links between plasma OAβ levels and memory performance in patients with AD. Using different methods, such as the enzyme-linked immunosorbent assay (ELISA), several studies have shown that OAβ levels in the plasma or serum were associated with general cognitive performance in patients with AD [17–19]. However, large variations in OAβ concentrations were observed across studies. Previously, Wang et al., Yang et al., An et al., and Youn et al. demonstrated that elevations in plasma OAβ could be monitored with the multimer detection system (MDS) [19–22]. However, the study based on the MDS has not been involved the clinical relevance of the plasma OAβ in AD.

Therefore, we designed this study on the basis of previous reports by Wang et al., Yang et al., An et al., and Youn et al. [19–22] to compare the levels of plasma OAβ between the AD and the control groups and to examine the associations between plasma OAβ levels and memory performance, including immediate and delayed recalls. We hypothesized that the plasma OAβ level was elevated in patients with AD and also correlated with the memory performance in patients with AD.

## Methods

### Participants

From March to September 2017, 30 people with AD and 28 cognitively normal controls were recruited from the case registry at the Dementia Care and Research Centre, Peking University Institute of Mental Health. All research participants were administered the neuropsychological test battery, underwent MRI scanning, and received a thorough blood test for measures including blood cell count, hepatic and renal function, thyroid function, and levels of vitamin B_12_ and folic acid, as well as serum syphilis testing. A dementia specialist interviewed all participants prior to making each diagnosis.

The inclusion criteria for the AD group were as follows: (1) were aged between 60 and 90 years; (2) met the criteria for dementia according to the International Classification of Diseases, 10th Revision (ICD-10) and the criteria for probable AD of the National Institute of Neurological and Communicative Disorders and the Stroke/Alzheimer Disease and Related Disorders Association (NINCDS-ADRDA); (3) had more than 6 years of education; and (4) had a score on the modified Hachinski ischemic scale of ≤ 4.

The inclusion criteria for the control group were as follows: (1) were aged between 60 and 90 years, (2) had more than 6 years of education, (3) did not have memory complaints, and (4) did not have detected cognitive impairment.

Individuals who had major medical problems, such as tumors, cerebrovascular events, or psychiatric disorders, such as depression, schizophrenia, or alcohol-related disorder, were excluded.

The study protocol was approved by the Institutional Review Board of Peking University Sixth Hospital. Informed consent was obtained from each participant.

### Neuropsychological assessment

Standardized procedures were used to administer the neuropsychological test battery as described previously [23]. Briefly, the overall cognitive function was assessed with the Mini-Mental State Examination (MMSE), Cognitive Abilities Screening Instrument (CASI), and Alzheimer’s Disease Assessment Scale–cognitive portion (ADAS-Cog) [24–26].

The neuropsychological test battery measured specific cognitive domains, including common objects memory test (COMT) for episodic memory; Stroop test for executive function; the animal naming test for language; read and set time, Consortium to Establish a Registry for Alzheimer’s Disease (CERAD) drawing and block design for visuospatial function; digit span (forward and backward) for attention; and picture completion for reasoning [27].

The present study paid particularly close attention to COMT performance. The COMT was developed to provide a culturally sensitive measure of recent memory, specifically for use in a cross-cultural neuropsychological test battery. This test was administered using standardized procedures, as previously described [27]. Briefly, the participants were shown a set of ten colored pictures of common objects (e.g., button, chair, scissors, clock, comb, cup, key, knife, leaf, and umbrella) during three learning trials at the rate of one picture per 2 s. The pictures were spiral-bound and presented in a standard order that differed among the three trials; the participants were required to immediately recall as many objects as possible after each learning trial. After all three trials, the participants engaged in brief distractor tasks (e.g., CERAD figure drawing) for 3 to 5 min and were subsequently asked again to recall the previous objects. This recall order was serially recorded. The 5-min delayed recall was immediately followed by a recognition test, in which ten original objects were interspersed with ten distractors. The subjects were asked to indicate with a simple “yes” or “no” whether an item was observed in the original three learning tests. The distractor objects were similar to the original objects in terms of the visual complexity and lacking distinctive details. Long-term retention of the original objects was assessed after a 30-min delay using tests of recall and recognition, with a different set of ten distractors.

### Plasma preparation

Samples were treated with heparin and EDTA to compare the effects of the two anticoagulants on the OAβ levels. Venous blood was collected in 6-ml BD Vacutainer® EDTA tubes and 10-ml BD Vacutainer® heparin tubes, followed by centrifugation at 850×*g* for 30 min at room temperature (RT). The separation of plasma was performed within 3 h of sample collection. The plasma was aliquoted into polypropylene tubes (1.5 ml) in volumes of 500 μl and stored at − 80 °C until assayed.

### Quantifying the levels of plasma OAβ

The MDS-AD assay kit (donated by the PeopleBio, Inc., Korea) was used to quantify the levels of OAβ in the plasma. The antibodies used in the assay kit were the mouse monoclonal antibody 6E10 (BioLegend, San Diego, CA, USA) and WO2-HRP antibody (Absolute Antibody Ltd., Oxford, UK). A well-trained technician was blind to the diagnostic information of the samples and performed the experiments according to the manufacturer’s protocol [19–22]. More details of the quality of assay are provided in Additional file 2: Table S1.

Prior to the procedure, aliquots of plasma samples were thawed at 37 °C for 15 min. Ten microliters of plasma, 4 μl of HAMA (human anti-murine antibody, HAMA) blocker (Scantibodies Laboratory, Santee, CA, USA), and 90 μl of assay buffer were mixed. Ten microliters of PBR-1 (1% proprietary + 1.25% dimethyl sulfoxide (DMSO) + 96.75% phosphate-buffered saline contains Tween 20 (PBST) + 1% ultra-pure water) was mixed into the plasma mixture. Then, the heparin-treated plasma mixtures and EDTA-treated plasma mixtures were incubated for 48 h and 1 h, respectively. The plasma sample mixture and serially diluted standards were added to separate wells of the plate in a total volume of 100 μl. The plates were incubated at RT for 1 h. After washing three times with washing buffer, the detection antibody was added to the wells, and the plate was incubated for 1 h at RT. Finally, 100 μl of 3, 3′, 5, 5′-tetramethylbenzidine (TMB) reagent was added as a substrate, and after 15 min, the reaction was stopped with 50 μl of 1 M H_2_SO_4_. Optical density (OD) values were measured at 450 nm using a Victor 3™ multi-spectrophotometer.

After the experiments, OD from the samples and the standard curve were used to calculate the levels of OAβ in the plasma. The analysis was performed for both ODs and absolute concentration which was converted from the ODs. We did the serially diluted standards for quality control. In our paper, *R*^2^ value of the standard curve was about 0.99 (shown in Additional file 1: Figure S1). In addition, we think it is better to present raw data other than the absolute concentrations in research papers. Until now, detecting crude oligomeric Aβ in the plasma was a challenge, owing to its low concentrations in the blood. MDS for AD was optimized to enhance detection by spiking synthetic Aβ_42_ into the plasma as mentioned in a recent study. Previous papers using the MDS test detected the raw luminescence signal and used relative luminescence units (RLU) to present the OAβ levels [19]. Therefore, we chose to present the ODs as the main results.

### Statistical analysis

Data analysis was performed with IBM SPSS Statistics 20.0 software. Student’s *t* tests were used to compare the ages and educational levels, and the two-tailed chi-square (*χ*^2^) tests were used to compare sex ratio and apolipoprotein Eε4 (APOEε4) status between the AD and control groups.

The levels of plasma OAβ and the cognitive performances between the two groups were compared with Student’s *t* tests. A linear regression model was used to analyze the associations between the plasma OAβ levels from the samples processed with the EDTA and heparin anticoagulants. Next, Pearson’s correlation analysis was performed to examine the associations between plasma OAβ levels and cognitive test scores. A partial correlation analysis adjusting for age, sex, educational level, and APOEε4 status was used in a subsequent investigation of the correlations. Potential group differences with respect to the COMT memory performances were investigated with analysis of variance (ANOVA) and ANOVA for repeated measures.

To explore the utility of OAβ in assisting the clinical diagnosis, we computed the area under the receiving operating characteristic (ROC) curve (AUC) that discriminated patients with AD from controls. All tests were two-sided. The statistical significance was set at *p* < 0.05.

## Results

### Demographic characteristics of the research participants

As shown in Table 1, the average age in the control group was 71.9 ± 7.2 and that in the AD group was 76.9 ± 5.8 (*p* = 0.006). No significant group difference was observed in the comparison of the sex ratio or in their educational levels (*p* > 0.05).

**Table 1:** Demographic characteristics of the study participants

	AD group (*n* = 30)	Control group (*n* = 28)	*t*/*χ*^2^	*p* value
Age (years), mean (SD)	76.9 (5.8)	71.9 (7.2)	− 2.88	0.006
Sex, M/F (*n*)	15/15	7/21	5.59	0.05
Education (years), mean (SD)	13.6 (2.7)	14.3 (1.9)	1.06	0.30
APOEε4 carriers (*n*)	16	5	7.89	0.005

The two-tailed chi-square (*χ*^2^) test was used to compare the distribution of sex and APOEε4 status in the two groups. Student’s *t* tests (*t*) were used for age and education

*AD* Alzheimer’s disease, *SD* standard deviation

### Comparison of cognitive performance in the AD and control groups

As expected, the AD group exhibited lower performance on overall cognitive function and the episodic memory test than the control group (all *p* < 0.001, Table 2).

**Table 2:** Cognitive performance in the AD and control groups

Cognitive measures	AD group (*n* = 30)	Control group (*n* = 28)	*p* value
MMSE	19.6 (5. 4)	28.4 (1.3)	< 0.001
CASI	72.3 (14.0)	94.2 (4.5)	< 0.001
ADAS-Cog	21.2 (10.4)	4.3 (2.8)	< 0.001
COMT			
IR-T1	3.1 (1.7)	6.7 (1.4)	< 0.001
IR-T2	4.4 (1.6)	8.4 (1.2)	< 0.001
IR-T3	5.2 (1.5)	9.0 (0.9)	< 0.001
DR5	2.6 (2.1)	8.4 (1.2)	< 0.001
DR30	1.8 (2.0)	8.6 (1.0)	< 0.001

Data are presented as the mean (SD). *p* values were obtained using Student’s *t* tests for all data

*AD* Alzheimer’s disease, *MMSE* Mini-Mental State Examination, *CASI* Cognitive Ability Screening Instrument, *ADAS-Cog* Alzheimer’s disease assessment scale–cognitive portion, *COMT* common object memory test, *IR-T1* trial #1 of the immediate recall test, *IR-T2* trial #2 of the immediate recall test, *IR-T3* trial #3 of the immediate recall test, *DR5* 5-min delayed recall test, *DR30* 30-min delayed recall test

**Fig. 1:**
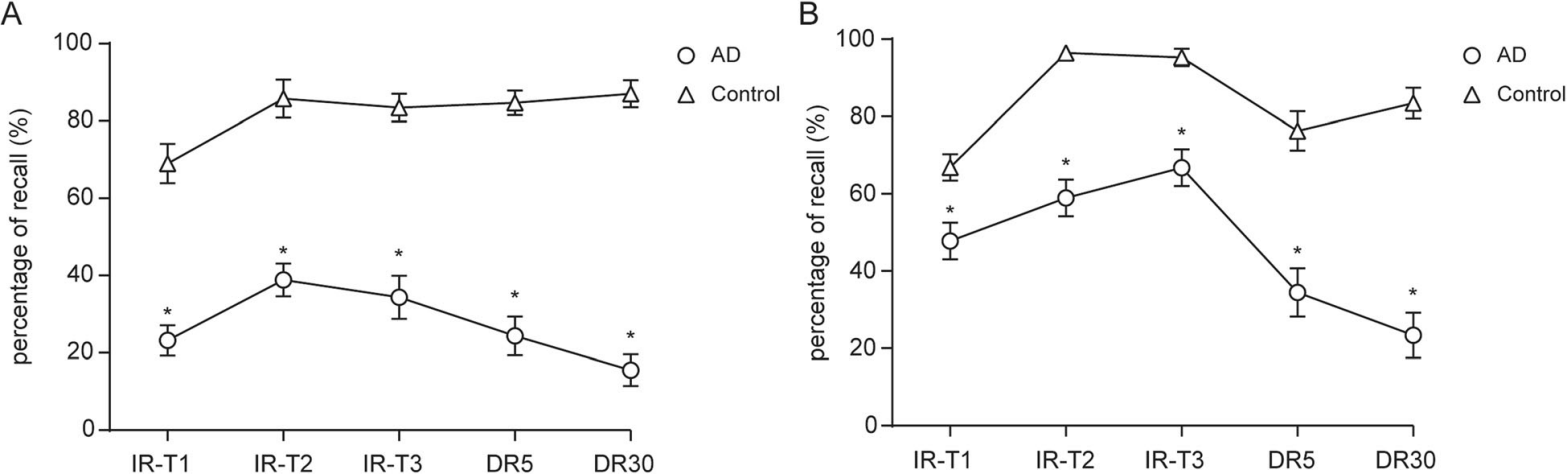
Common objects memory test (COMT) results for patients with Alzheimer’s disease (AD) compared with normal controls. The recall percentage for the first three words (**a**) and the last three words (**b**) on the wordlist was lower in the AD patients than in the normal controls. IR-T1, trial #1 of the immediate recall test; IR-T2, trial #2 of the immediate recall test; IR-T3, trial #3 of the immediate recall test; DR5, 5-min delayed recall test; DR30, 30-min delayed recall test. *p < 0.05

Specifically, the AD group presented a significant decrease in the number of recalled items immediately or after the 5- and 30-min delays (*p* < 0.001, Table 2). Furthermore, recall of the first three words (primacy effect) (Fig. 1a) and the last three words (recency effect) (Fig. 1b) of the COMT wordlist was lower in the AD group than in the control group (Fig. 1).

### Differences in plasma OAβ OD values between the two groups

**Fig. 2:**
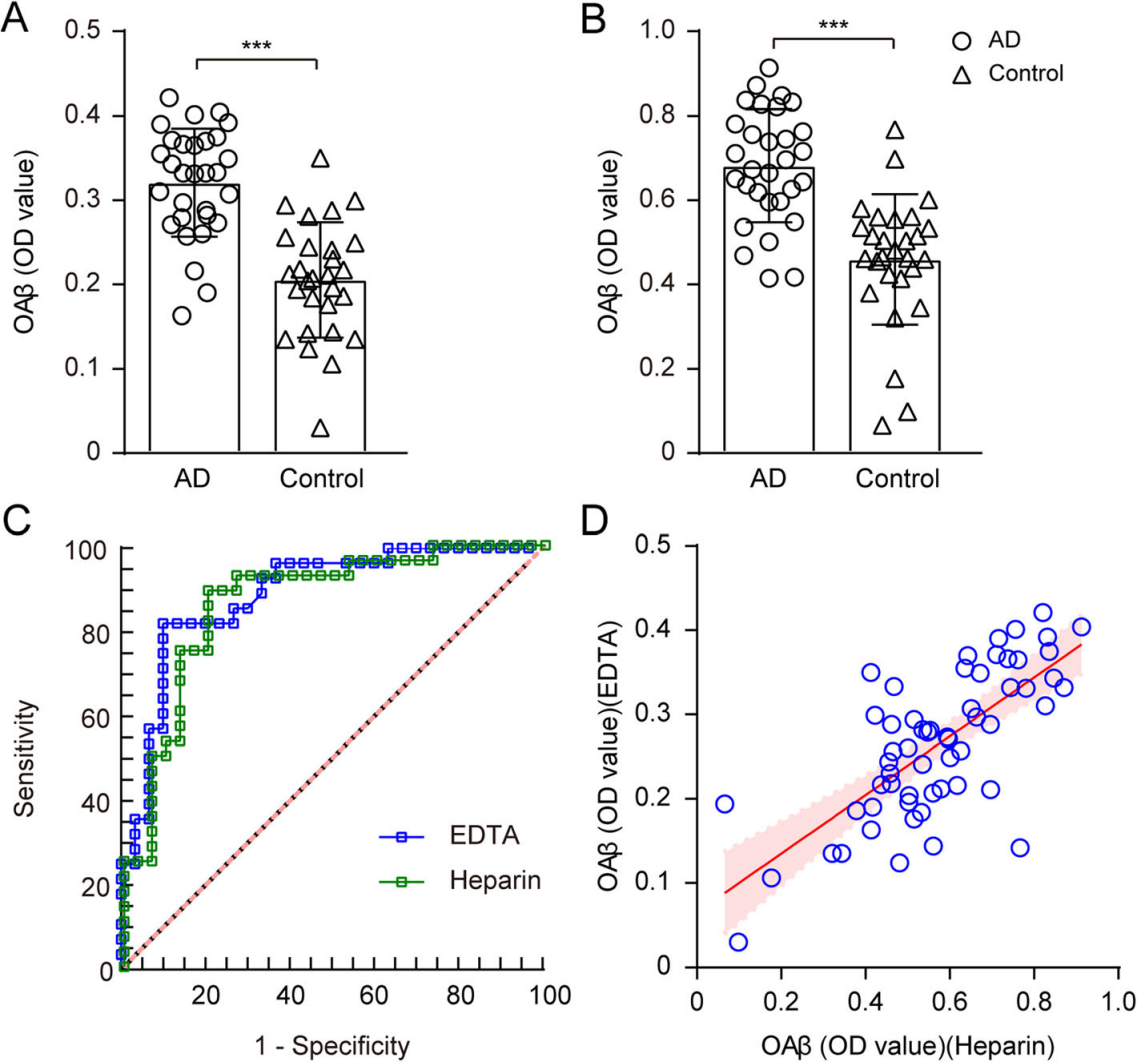
Effect of anticoagulants on OAβ OD values in the plasma. Comparison of the OAβ levels in patients with AD and in normal controls from samples processed with EDTA (**a**) and heparin (**b**) anticoagulants. **c** ROC analysis of the plasma OAβ OD values measured using the MDS. **d** The scatter plot shows the linear regression between the OAβ OD values in the plasma processed with the anticoagulant heparin (*x*-axis) and EDTA (*y*-axis). The shaded area shows the 95% CI

The plasma OAβ OD values were markedly elevated in the AD group compared with the control group (*p* < 0.001 for samples anti-coagulated with either EDTA or heparin (Fig. 2a, b). The EDTA plasma Aβ oligomer OD value measured using the MDS could discriminate between the AD and control groups with an AUC of 0.89. The best sensitivity and specificity levels were 82.1% and 90.0%, respectively (Fig. 2c). With the heparin-treated samples, the MDS also showed a high sensitivity of 75.0% and specificity of 86.7% between the AD and control groups with an AUC of 0.87 (Fig. 2c).

**Fig. 3:**
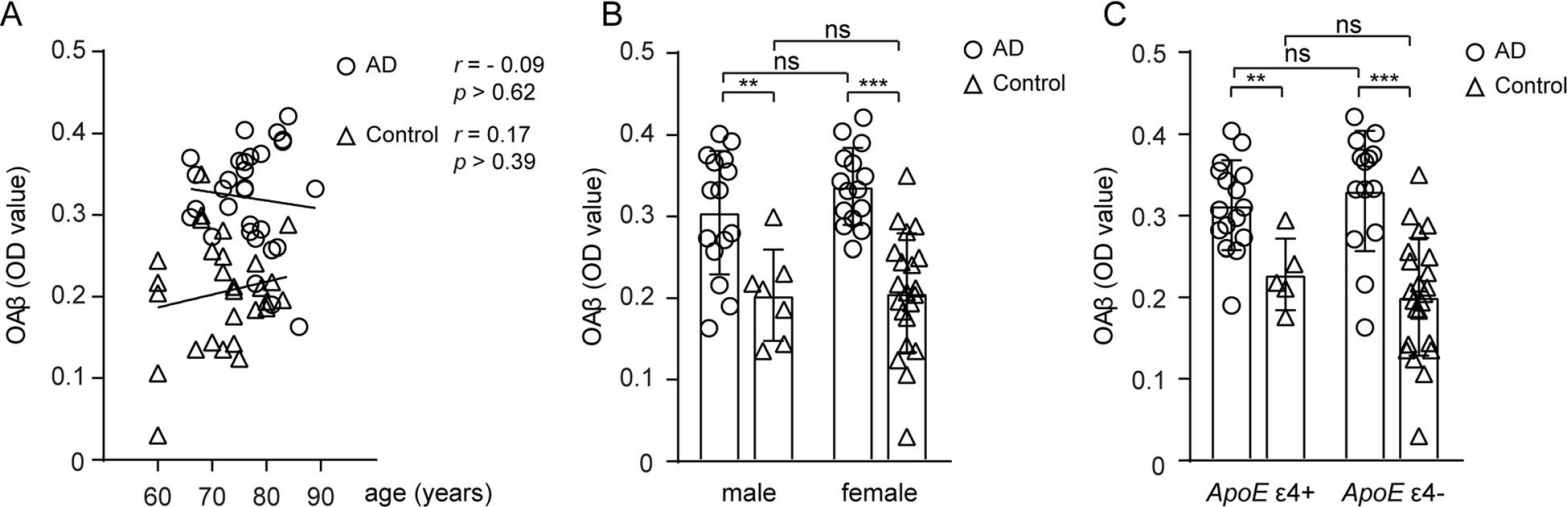
The effects of age, sex, and ApoEε4 status on the plasma OAβ OD values in the AD (circle) and control (triangle) groups. **a** The plasma OAβ levels were not correlated with age (*p* > 0.05). **b** There was no significant difference in the plasma OAβ levels between men and women. **c** There was no significant difference in the plasma OAβ levels between APOEε4 carriers and non-carriers. AD, Alzheimer’s disease

The levels of OAβ in the EDTA-treated plasma showed a strong direct correlation with the heparin-treated plasma (*r* = 0.35, *p* < 0.001, see Fig. 2d). Hereafter, the experiment was carried out using EDTA, which is more commonly used. All of the following data were analyzed using the levels of OAβ OD in the EDTA-treated plasma. There was no effect of age, sex, or ApoE4 status on the plasma OAβ OD values (*p* > 0.05, Fig. 3).

The inter-group difference in the plasma OAβ levels remained significant (*p* < 0.05) after taking age into account. This result was replicated in the heparin- and EDTA-treated plasma samples.

### Correlation of plasma OAβ levels and overall cognitive function

**Fig. 4:**
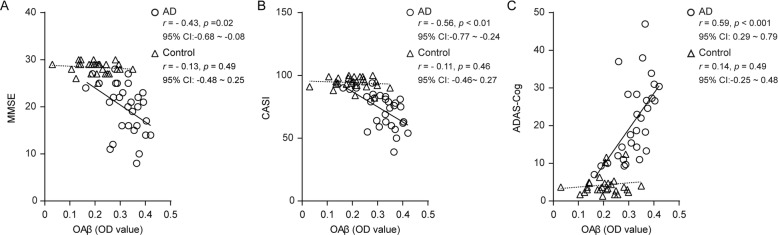
The plasma OAβ OD values were significantly correlated with cognitive function. The scatter plots show the correlation of the plasma OAβ levels with the scores on the MMSE (**a**), CASI (**b**), and ADAS-Cog (**c**) tests in the AD (circle and solid line) and control (triangle and dotted line) groups. AD, Alzheimer’s disease; MMSE, Mini-Mental State Examination; CASI, Cognitive Ability Screening Instrument; ADAS-Cog, Alzheimer’s disease assessment scale–cognitive portion

The plasma OAβ OD value correlated strongly with the scores on the MMSE (*r* = − 0.43, *p* = 0.02), CASI (*r* = − 0.56, *p* < 0.01), and ADAS-Cog (*r* = − 0.59, *p* < 0.001) in the AD group (Fig. 4). After adjusting for age, sex, educational status, and ApoEε4 status, the linear regression models preserved the significant direct correlation between the plasma OAβ OD values and overall cognitive function (Additional file 3: Table S2). In comparison, no significant association between the OAβ OD values and cognitive test scores was observed in the control group (*p* > 0.05).

### Correlation of the plasma OAβ OD values with episodic memory

**Fig. 5:**
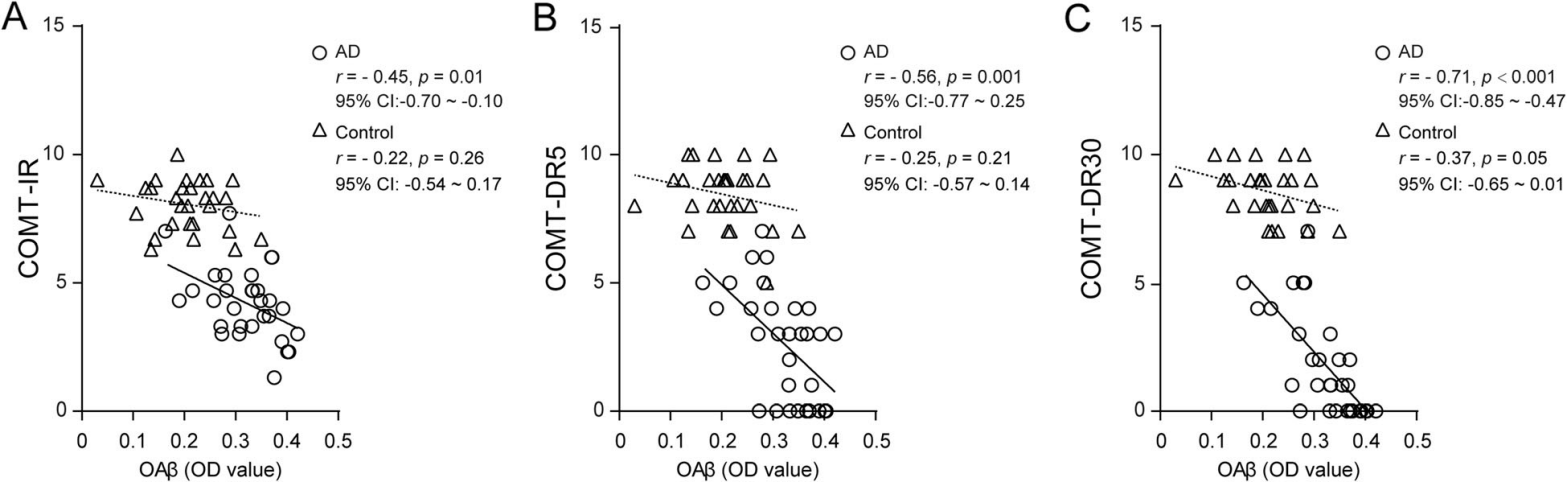
The plasma OAβ OD values were significantly correlated with the COMT scores in patients with AD and normal controls. The scatter plots show the correlation of the OAβ levels with the immediate (**a**), 5-min (**b**), and 30-min (**c**) delayed recall scores on the COMT in the AD (circle and solid line) and control (triangle and dotted line) groups. COMT, common object memory test; COMT-IR, immediate recall of the COMT; COMT-DR5, 5-min delayed recall of the COMT; COMT-DR30, 30-min delayed recall of the COMT

In the AD group, significant associations were noticed between the plasma OAβ levels and COMT immediate (*r* = − 0.45, *p* = 0.01), 5-min (*r* = − 0.56, *p* < 0.01) and 30-min (*r* = − 0.71, *p* < 0.001, Fig. 5) recall scores. The statistical significance remained after adjusting for age, sex, education, and ApoEε4 status (Table 3). On the other hand, no significant associations were observed between the plasma OAβ levels and the primacy and recency effects in the memory test (*p* > 0.05).

**Table 3:** Correlations of plasma OAβ levels with the immediate and delayed recall scores on the COMT after adjusting for age, sex, educational level, and ApoEε4 status

	AD (*n* = 30)		Control (*n* = 28)	
	*r*	*p*	*r*	*p*
Episodic memory				
COMT-IR	− 0.60	0.002	− 0.13	0.56
COMT-DR5	− 0.54	0.006	− 0.18	0.41
COMT-DR30	− 0.71	< 0.001	− 0.23	0.27

*AD* Alzheimer’s disease, *COMT* common object memory test, *IR* immediate recall (average of three trials), *DR5* 5-min delayed recall, *DR30* 30-min delayed recall

In the control group, a weak associative trend was observed only between the plasma OAβ OD values and the 30-min delayed recall scores (*p* = 0.05) (Fig. 5c). No significant correlation was observed between the plasma OAβ levels and any other memory measure.

## Discussion

In the present study, the levels of plasma OAβ were elevated in patients with AD compared to controls. Interestingly, the elevated plasma levels of OAβ strongly correlated with the decreased general cognitive level and deteriorated episodic memory performance in patients with AD in comparison with healthy age-matched normal subjects. The current observations support the notion that plasma OAβ could serve as a potential blood-based biomarker for the early diagnosis of AD.

OAβ have been reported as the most neurotoxic agents in the pathological process of AD [6]. Previous studies have revealed that soluble OAβ in the brain were involved in the early stages of AD pathology and predate classic fibrillar amyloid plaque deposition, neuronal cell loss, and memory impairment [28]. Other previous studies have reported elevations in OAβ levels in CSF and brain tissue in patients with AD [8, 29, 30]. Wang et al. demonstrated that the levels of plasma OAβ were associated with CSF levels of Aβ_42_, phosphorylated tau protein (pTau), and total tau protein (tTau) [19]. The positive correlation between the levels of OAβ detected in serum and those in matched samples of CSF has been found by Kasai et al. The result suggested that OAβ could diffuse or be efficiently transported across the blood–brain or the blood–CSF barrier (collectively referred to as the BBB) and/or that the BBB allowed Aβ carrier proteins to be transferred between the blood and CSF, which would transfer Aβ peptides to the blood in the form of OAβ complexes [31]. Our study provided the support that the levels of OAβ in the plasma were increased in the patients with AD, which was consistent with previous reports, and therefore, the increased plasma levels of OAβ could be indicative of AD pathology.

One of the most exciting findings from the present study was that the plasma levels of OAβ were highly correlated with episodic memory performance (measured with the COMT) in addition to general cognitive function (measured with the MMSE, CASI, and ADAS-Cog). Previous studies have demonstrated that the level of soluble OAβ was correlated with the extent of synaptic loss, which would restrict hippocampal function [28]. Increased levels of OAβ were also described by Fukumoto et al. The levels of OAβ in AD or MCI patients has been found were significantly higher than in normal controls and correlated inversely with MMSE score. The AUC for the OAβ (0.844) was greater than that for the CSF Aβ_42_ (0.712), suggesting that OAβ may serve as a test for discriminating between AD/MCI patients and cognitively normal control [32]. The present study could be the first to report the correlation between plasma OAβ and episodic memory. Changes in episodic memory were reported as one of the earliest pathological features of AD. In various learning and recall paradigms, a delayed recall was considered the measurable characteristic of mild cognitive impairment and AD [24–26]. As a test with minimal cultural bias, the COMT has been widely used in cross-cultural assessments of memory performance [27]. The strong correlations between the plasma levels of OAβ and the immediate and delayed recall scores on the COMT revealed that the increased levels of plasma OAβ paralleled the earliest cognitive changes in patients with AD. Coupled with the relevance to the severity of dementia, the significance of plasma OAβ in accordance with the earliest cognitive changes could present additional evidence in support of plasma OAβ as a potential blood-based biomarker for AD screening or diagnosis.

Furthermore, this study demonstrated the reliability of quantifying the OAβ in the plasma. In previous studies by Wang et al., Yang et al., An et al., and Youn et al., the MDS method was applied to the plasma samples with heparin as an anticoagulant [19–22]. The present study found that the levels of OAβ in the EDTA-treated plasma samples were highly correlated with those in the heparin-treated plasma samples, indicating that OAβ levels are relatively stable and independent, allowing reliable quantification in the plasma samples treated with either EDTA or heparin anticoagulant.

Although the present study revealed the interesting result of OAβ levels as a potential blood-based biomarker, our findings should be interpreted with caution and have the following limitations: First, the study was performed with samples collected from a cross-sectional study. Future longitudinal studies are warranted to investigate and verify whether plasma OAβ could monitor and/or represent disease progression. The trajectory of the OAβ levels in the plasma could provide evidence on its clinical application as a prognostic biomarker [28–30]. Second, we did not replicate the analysis on the correlation between plasma oligomer and CSF AD biomarkers reported by Wang et al. [19]. However, according to the five-phase model of biomarker discovery proposed by Frisoni et al. [33], it is even more important to examine the evidence of the clinical relevance of the plasma oligomer in AD. Our exploratory clinical assay would support future studies that will use a large sample of longitudinal data available in the repositories to explore the usefulness of plasma oligomer in the detection of AD. Third, examining the profile of MCI would strengthen the utility of oligomer as a potential biomarker for AD. Our study was designed on the basis of the previous report by Wang et al. and aimed to examine whether the level of oligomer was associated with the memory performance [19]. A significant association indicated, to some extent, the level of oligomer might reflect the clinical phenotype. In addition, the lack of patients with neurodegenerative diseases other than AD, such as frontotemporal dementia and dementia of Lewy body, would prevent our testing for disease specificity of plasma OAβ. To the best of our knowledge, the evidence of the blood-based biomarkers of AD remains limited, especially when discriminating AD from other neurodegenerative diseases. Our study explored the potential of OAβ in the plasma to differentiate AD from normal cognition. It remains further investigations to establish its usefulness in screening for dementia. The MDS assay remained in the early phase of development. Additional retrospective studies with a larger sample size would be needed. Such studies have recently been planned in Asian and European countries. Additionally, further studies are needed for addressing the role of plasma OAβ in AD detection from both neuropathological and clinical perspective. Prospective studies on the diagnostic accuracy with confirmed neuropathological cases, via MRI, amyloid PET, or CSF biomarkers, should be considered for validating the application of plasma OAβ levels as a diagnostic biomarker.

## Conclusion

The present study indicates that elevated levels of OAβ in the plasma, which strongly correlate with symptom severity, especially episodic memory, might be a potential biomarker for AD diagnosis. Since plasma samples are easily obtained and MDS is simple to perform, measuring OAβ in the plasma could be a potential diagnostic screening approach in the clinical setting once the cutoff value is determined.

## Additional files

Additional file 1: **Figure S1.** The best fit curve plotting the absorbance value (OD value, Y axis) against the absolute concentration (X axis). The standard curve equation is Y = 0.1618X + 0.0078. For example, if OD value of sample A is 0.25, substitute Y with 0.25 and solve for the concentration value of X in the equation. X = (0.25 –0.0078)/0.1618 = 1.49 ng/ml. (PDF 105 kb)

Additional file 2: **Table S1.** Information of the quality of the MDS assay. (DOCX 14 kb)

Additional file 3: **Table S2.** Correlation of plasma OAβ OD values with overall cognition after adjusting for age, sex, educational level and APOE ε4 status. (DOCX 22 kb)

## Data Availability

The dataset generated and analyzed during the current study is not publicly available because we are preparing an additional manuscript. However, they are available upon reasonable request to the corresponding authors.

## Abbreviations

AD: Alzheimer’s disease
Aβ: Amyloid-β
OAβ: Oligomeric amyloid-β
ADAS-Cog: Alzheimer’s Disease Assessment Scale–cognitive portion
APOE: Apolipoprotein E
ANOVA: Analysis of variance
AUC: Area under the curve
CSF: Cerebrospinal fluid
CASI: Cognitive Abilities Screening Instrument
COMT: Common Objects Memory Test
CERAD: Consortium to Establish a Registry for Alzheimer’s Disease
ELISA: Enzyme-linked immunosorbent assay
HAMA: Human anti-murine antibody
IR-T1: Trial #1 of the immediate recall test
IR-T2: Trial #2 of the immediate recall test
IR-T3: Trial #3 of the immediate recall test
DR5: 5-min delayed recall test
DR30: 30-min delayed recall test
MDS: Multimer detection system
MMSE: Mini-Mental State Examination
OD: Optical density
pTau: Phosphorylated tau protein
RT: Room temperature
ROC: Receiving operating curve
RLU: Relative luminescence units
TMB: Tetramethylbenzidine
tTau: Total tau protein
