# Supplementary figures and images for: Association between increased levels of amyloid-β oligomers in plasma and episodic memory loss in Alzheimer’s disease
Source: Alzheimers Res Ther. 2019 Oct 25;11:89. doi: 10.1186/s13195-019-0535-7 (PMC6814096; doi:10.1186/s13195-019-0535-7)

OD values

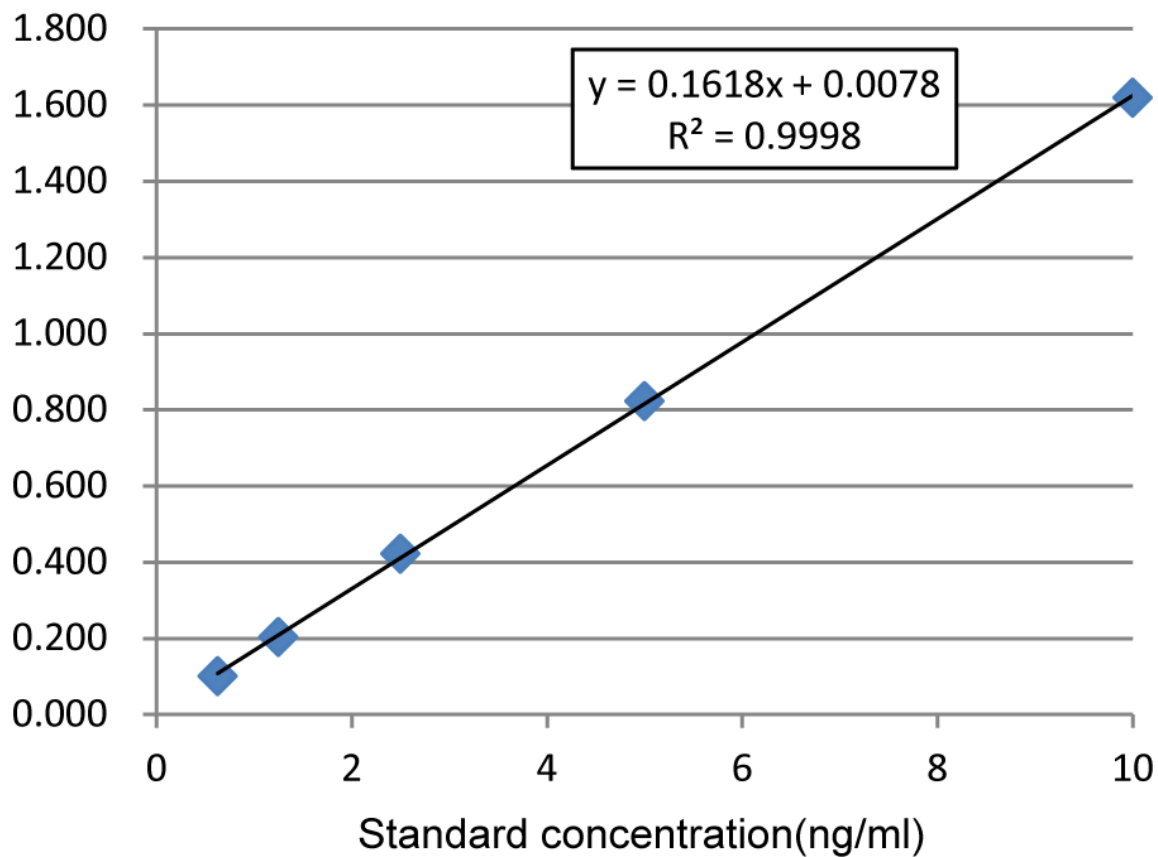

Supplement: Supplementary file 1 — Figure S1. The best fit curve plotting the absorbance value (OD value, Y axis) against the absolute concentration (X axis). The standard curve equation is Y = 0.1618X + 0.0078. For example, if OD value of sample A is 0.25, substitute Y with 0.25 and solve for the concentration value of X in the equation. X = (0.25 –0.0078)/0.1618 = 1.49 ng/ml. (PDF 105 kb) [file 13195_2019_535_MOESM1_ESM.pdf]
