# Supplementary material for: Association between increased levels of amyloid-β oligomers in plasma and episodic memory loss in Alzheimer’s disease
Source: Alzheimers Res Ther. 2019 Oct 25;11:89. doi: 10.1186/s13195-019-0535-7 (PMC6814096; doi:10.1186/s13195-019-0535-7)
Supplement: Supplementary file 2 — Table S1. Information of the quality of the MDS assay. (DOCX 14 kb) [file 13195_2019_535_MOESM2_ESM.docx]

Table S1. Information of the quality of the MDS assay

| Measurement | Findings |
| --- | --- |
| *Pre-analytical* |  |
| Sample storage stability | Length of storage stability is unknown but fresh plasma yields better stability. |
| Sample handling stability (freeze-thaw cycles) | < 3 |
| Recovery (if extraction involved) | 118.7% (ELISA spike recovery) |
| Interfering substances/conditions? | None |
| *Analytical* | |
| Limit of blank | 0.371 ng/mL |
| Limit of detection | 0.377 ng/mL |
| Precision repeatability (intra-assay %CV) | 18.69% |
| Precision repeatability (inter-assay %CV) | 12.13% |
| Specificity (Cross Reactivity) | No cross-reactivity from peptides with similar structure (tested for: Aβ_1-42_ monomer; Aβ_4-42_; Aβ_9-42_; and Aβ_1-24_ peptide) |
| Selectivity | No interferences from hemoglobin, bilirubin, albumin, cholesterol, heparin |
| Quality Control processes | Yes (Run rejection criteria) |
| External Proficiency (Alternatives) | No |
| Normal range for healthy adult (if known) | Unknown |
