# Supplementary material for: Association between increased levels of amyloid-β oligomers in plasma and episodic memory loss in Alzheimer’s disease
Source: Alzheimers Res Ther. 2019 Oct 25;11:89. doi: 10.1186/s13195-019-0535-7 (PMC6814096; doi:10.1186/s13195-019-0535-7)
Supplement: Supplementary file 3 — Table S2. Correlation of plasma OAβ OD values with overall cognition after adjusting for age, sex, educational level and APOE ε4 status. (DOCX 22 kb) [file 13195_2019_535_MOESM3_ESM.docx]

**Table S2**. Correlation of plasma OAβ levels with overall cognition after adjusting for age, sex, educational level and ApoE ε4 status

|  | AD (n=30) | |  | Control (n=28) | |
| --- | --- | --- | --- | --- | --- |
|  | *r* | *p* |  | *r* | *p* |
| MMSE | -0.45 | 0.03 |  | -0.08 | 0.72 |
| CASI | -0.50 | 0.01 |  | 0.03 | 0.89 |
| ADAS-Cog | 0.64 | 0.001 |  | -0.13 | 0.55 |

AD: Alzheimer’s disease; MMSE: Mini-Mental State Examination; CASI: Cognitive Ability Screening Instrument; ADAS-Cog, Alzheimer’s disease assessment scale–cognitive portion**.**
